# Risk of gallstones based on ABCG8 rs11887534 single nucleotide polymorphism among Taiwanese men and women

**DOI:** 10.1186/s12876-021-02060-5

**Published:** 2021-12-14

**Authors:** Keng-Wei Liang, Hsin-Hui Huang, Lee Wang, Wen-Yu Lu, Ying-Hsiang Chou, Disline Manli Tantoh, Oswald Ndi Nfor, Neng-Yu Chiu, Yeu-Sheng Tyan, Yung-Po Liaw

**Affiliations:** 1 Institute of Medicine, Chung Shan Medical University, Taichung City, 40201 Taiwan; 2 School of Medicine, Chung Shan Medical University, Taichung, 40201 Taiwan; 3 Department of Medical Imaging and Radiological Sciences, Chung Shan Medical University, Taichung, 40201 Taiwan; 4 Department of Medical Imaging, Chung Shan Medical University Hospital, Taichung City, 40201 Taiwan; 5 Department of Public Health and Institute of Public Health, Chung Shan Medical University, Taichung, 40201 Taiwan; 6 Department of Radiation Oncology, Chung Shan Medical University, Taichung, 40201 Taiwan

**Keywords:** ABCG8, rs11887534, Dominant model, Gallstones, Hormone use

## Abstract

**Background:**

Gallstones are abnormal masses caused by impaired metabolism of cholesterol, bilirubin, or bile salts in the gallbladder or biliary tract. ATP-binding cassette subfamily G member 8 (ABCG8) is a protein that regulates cholesterol efflux from the liver. Genome-wide association studies (GWAS) and meta-analyses of GWAS revealed the ABCG8 rs11887534 variant as the most common genetic determinant of gallstones in humans. These findings have not been extensively replicated in Taiwanese. Therefore, we appraised the relationship between gallstones and rs11887534 in a relatively large Taiwanese sample.

**Methods:**

We retrieved data collected through questionnaires, physical and biochemical tests from the Taiwan Biobank Bank (TWB). The study participants comprised 7388 men and 13,880 women who voluntarily enrolled in the Taiwan Biobank project between 2008 and 2019. Gallstones were self-reported.

**Results:**

The overall sample size was 21,268 comprising 938 gallstone patients and 20,330 non-gallstone individuals. Among the participants, 20,640 had the GG and 628 had the GC + CC genotype. At *p*-value < 0.05, the baseline genotypes and gallstone status between men and women were not significantly different. The risk of gallstones was higher in participants having the GC + CC compared to the GG genotype: odds ratio (OR); 95% confidence interval (CI) = 1.698; 1.240–2.325), but was lower in men compared to women (OR = 0.763; 95% CI = 0.638–0.913). Compared to men with the rs11887534 GG genotype, women with the GG and GC + CC genotypes had a higher risk of gallstone (OR; 95% CI = 1.304; 1.087–1.565 for GG and 2.291; 1.514–3.467 for GC + CC). The positive association between GC + CC and gallstones was retained after we restricted the analysis to the female participants (OR; 95% CI = 1.789 = 1.208–2.648). Hormone use was associated with an elevated risk of gallstones (OR; 95% CI = 1.359; 1.107–1.668). Relative to GG and no hormone use, we found a significantly high risk among hormone users with the GC + CC genotype (OR; 95% CI = 3.596; 1.495–8.650).

**Conclusions:**

The rs11887534 GC + CC genotype was independently associated with a higher risk of gallstones. This risk was much higher among women, especially those who used hormones for various gynecological purposes.

**Supplementary Information:**

The online version contains supplementary material available at 10.1186/s12876-021-02060-5.

## Introduction

Gallstones are characterized by abnormal (solid, pebble-like) masses composed of excessive cholesterol, bilirubin, or bile salts in the gallbladder [1]. Gallstone disease, the most common biliary system disorder is a risk factor for gallbladder cancer which significantly contributes to health care burdens [1]. The burden of gallstone disease in Taiwan is increasing and so, the disease is becoming a public health concern in the country [2]. In Taiwan, the prevalence of gallstones was 4.3% in 1989 [3] and 10.7% in 1995 [4] implying a 6.4% difference within this time interval. Huang and his team conducted a nationwide study between 1997 and 2005 and reported a rise in the rate of hospitalization for severe gallstone disease [5]. A cross-sectional study in a rural community reported a 5% prevalence of gallstones between 2003 and 2004 [6] while a hospital-based study found a 7.6% prevalence in 2005 [7]. Furthermore, a cohort study conducted between 2002 and 2007 found a 0.632% yearly incidence of gallstones [8].

Gallstone disease is a multifactorial disorder, involving an interplay between genetic and non-genetic factors. Some risk factors are female sex, older age, higher BMI, and family history [2, 6, 8–10]. Exogenous female sex hormones which contain estrogens are key players in the etiology of gallstones [11, 12]. For instance, increased contraceptive use and hormone replacement therapy confer increased susceptibility to gallbladder diseases [11–16]. Twin and family studies reported about 25–29% genetic contribution to gallstones [17, 18]. ATP-binding cassette transporter (ABCG8) which regulates cholesterol efflux from the liver has been reported as the main genetic susceptibility factor for gallstones [17, 19–21].

The ABCG8 rs11887534 polymorphism (a G to C change), also called D19H, is a missense variation where the negatively charged amino acid, histidine, substitutes the positively charged aspartic acid [22]. GWAS found the ABCG8 rs11887534 variant as the most common genetic determinant in humans [22–25]. It is important to validate these findings in people of different ancestry because genetic association studies are population-specific. Numerous replication studies confirmed the role of rs11887534 in gallstone formation [7, 22, 26–30]. Moreover, several meta-analyses confirmed this variant as the most common genetic determinant in humans with different ancestries [22, 23, 31, 32]. To our knowledge, only one study underscored the impact of rs11887534 on gallstone susceptibility among Taiwanese. However, only 979 individuals were included in the analysis [7]. A recent study recommended more research on rs11887534 polymorphism in relation to gallstones [21]. Moreover, the association between ABCG8 rs11887534 polymorphism according to sex has not been widely investigated. Taking into account the above concerns, we conducted this study to assess the risk of gallstones according to the ABCG8 rs11887534 polymorphism in a relatively large study sample involving Taiwanese men and women.

## Methods

### Taiwan biobank

We obtained data from the Taiwan biobank containing information on participants enrolled between 2008 and 2019. All the study participants met the eligibility criteria: strictly Taiwanese between aged 30 and 70 years with no prior cancer diagnosis. Each participant signed an informed consent letter before engaging in the data collection process, which was carried out in three phases: (1) self-responses to questionnaires (2) physical examination, and (3) biospecimen collection and examination [33]. We analyzed data from 21,268 participants (7388 men and 13,880 women) in the current study. Our work was approved by the Chung Shan Medical University Institutional Review Board (CS2-21005).

### ABCG8 rs11887534 genotyping

SNPs were genotyped on the TWB1.0 and TWB2.0 custom chips and imputed using the IMPUTE2 (v2.3.1) software. Details on SNP genotyping and imputation in the Taiwan Biobank have been previously described [34]. Summarily, the TWB1.0 chip contains SNPs typed on the Axiom Genome-Wide CHB 1 Array plate (Affymetrix, Inc., Santa Clara, CA, USA). The chip contains about 650,000 SNPs for GWAS. The TWB2.0 SNP chip (Thermo Fisher Scientific, Inc., Santa Clara, CA, USA) can identify approximately 714,431 SNPs. For quality assurance, SNPs were excluded from the Biobank for the following reasons: (1) call rate < 0.98, (2) heterozygosity rate > 5 standard deviation (SD), (3) from third-degree relatives and (4) from non-East Asians. The ABCG8 rs11887534 SNP was used in this study because it met the quality control criteria stated above.

### Variable definition

We obtained information on gallstone (yes/no), age, regular exercise (yes/no), cigarette smoking (yes/no), alcohol intake (yes/no), family history of gallstones (yes/no), sex (men/women), diabetes (yes/no), dietary fat, menopause (yes/no), and hormone use (yes/no) from the self-responses of the participants to the Taiwan biobank questionnaires.

In the questionnaires, participants were asked if they had a personal history of clinically confirmed gallstones. Family history of gallstones was confirmed if the participants’ father or mother has ever had gallstones. Hormone use was defined as the habit of using western hormonal medications to handle gynecological issues including menopause and contraception, among others for more than six months. In addition to self-reports, fasting glucose > 126 mg/dL or HbA1c ≥ 6.5% confirmed the presence of diabetes. Details on the other covariate (menopause, cigarette smoking, alcohol consumption, and regular exercise) assessments have been described elsewhere [35].

Body mass index (BMI) was grouped into four strata: normal BMI (18.5 ≤ BMI < 24 kg/m^2^), underweight (BMI < 18.5 kg/m^2^), overweight (24 ≤ BMI < 27 kg/m^2^), and obesity (BMI ≥ 27 kg/m^2^) according to the Taiwan Health Promotion Administration. The dietary fat score was derived from a food frequency questionnaire containing seventeen questions related to the intake of fat-rich foods in the previous month. The score was between 17 (low) and 85 (high) based on the sum of the responses to the 17 questions. The responses were ranked from 1–5: 1 (never), 2 (seldom), 3 (sometimes), 4 (frequently), and 5 (always). Levels of bilirubin, aspartate aminotransferase (AST), alanine transaminase (ALT), high-density lipoprotein cholesterol (HDL-C), low-density lipoprotein cholesterol (LDL-C), and triglyceride (TG) were determined at Chang Gung Memorial Hospital, Linkou, Taiwan using the Hitachi LST008 clinical analyzer (Tokyo, Japan).

### Statistical analysis

We used SAS version 9.4 (SAS Institute, Cary, NC, USA) and Plink 1.9 for data analysis. Baseline statistics in the male and female participants were obtained using chi-squared (χ2) and t-tests. Specifically, the differences in categorical data between the male and female participants were determined with the χ2 test while the differences in continuous data were determined with the t-test. Categorical data were presented as n (%) while continuous data were presented as mean ± standard error (SE). A *p*-value < 0.05 was set as the threshold for statistical significance. The association of gallstones with rs11887534 and other variables was determined by logistic regression analysis. The results were reported as odds ratios and 95% confidence intervals.

## Results

Table 1 describes the baseline profiles of participants by sex (7388 men and 13,880 women). The participants comprised 938 gallstone patients and 20,330 non-gallstone individuals. Among the participants, 20,640 had the GG and 628 had the GC + CC genotype. At baseline, genotypes, gallstone status, and age were not significantly different between the male and female participants (*p*-value = 0.1043, 0.4880, and 0.4702, respectively). The other variables were significantly different (*p* < 0.05) between men and women. Additional file 1: Table S1 shows the frequency of the GG, GC, and CC genotypes.

**Table 1:** Baseline profiles of the study participants stratified by sex

Variables	Women (n = 13,880)	Men (n = 7388)	*P*-value
Gallstone			0.1043
No	13,291 (95.76)	7039 (95.28)	
Yes	589 (4.24)	349 (4.72)	
ABCG8 rs11887534 genotype			0.4880
GG	13,462 (96.99)	7178 (97.16)	
GC + CC	418 (3.01)	210 (2.84)	
Age (years)	49.756 ± 0.088	49.868 ± 0.128	0.4702
Regular exercise			0.0023
No	8032 (57.87)	4115 (55.70)	
Yes	5848 (42.13)	3273 (44.30)	
Cigarette smoking			< .0001
No	13,272 (95.62)	4149 (56.16)	
Yes	608 (4.38)	3239 (43.84)	
Alcohol drinking			< .0001
No	13,571 (97.77)	5992 (81.10)	
Yes	309 (2.23)	1396 (18.90)	
BMI (kg/m^2^)			< .0001
18.5 ≤ BMI < 24 (Normal BMI)	7944 (57.23)	2666 (36.09)	
BMI < 18.5 (Underweight)	531 (3.83)	83 (1.12)	
24 ≤ BMI < 27 (Overweight)	3280 (23.63)	2698 (36.52)	
BMI ≥ 27 (Obesity)	2125 (15.31)	1941 (26.27)	
Family history of gallstones			0.0153
No	12,812 (92.31)	6887 (93.22)	
Yes	1068 (7.69)	501 (6.78)	
Diabetes			< .0001
No	12,759 (91.92)	6390 (86.49)	
Yes	1121 (8.08)	998 (13.51)	
Total bilirubin (mg/dL)	0.6138 ± 0.002	0.7516 ± 0.004	< .0001
AST (U/L)	23.1276 ± 0.100	26.2884 ± 0.173	< .0001
ALT (U/L)	20.8680 ± 0.147	29.9571 ± 0.274	< .0001
HDL-C (mg/dL)	58.3463 ± 0.113	48.1481 ± 0.131	< .0001
LDL-C (mg/dL)	120.7000 ± 0.271	122.3000 ± 0.368	0.0003
TG (mg/dL)	102.3000 ± 0.608	137.000 ± 1.288	< .0001
Dietary fat score	41.7411 ± 0.078	45.9518 ± 0.111	< .0001
Menopause			
No	6937 (49.98)	–	–
Yes	6943 (50.02)	–	–
Hormone use			
No	11,721 (84.45)	–	–
Yes	2159 (15.55)	–	–

Continuous and categorical data are presented as mean ± standard error (SE) and n (%), respectively

n = sample size, *ABCG8* ATP-binding cassette subfamily G Member 8, *BMI* body mass index, *AST* aspartate aminotransferase, *ALT* alanine transaminase, *HDL-C* high-density lipoprotein cholesterol, *LDL-C* low-density lipoprotein cholesterol, *TG* triglyceride

In the main effect model (Table 2), rs11887534 GC + CC compared to GG was associated with a higher risk of gallstone (OR = 1.698; 95% CI = 1.240–2.325). Men had a lower risk of gallstones than women (OR = 0.763; 95% CI = 0.638–0.913). Other factors which were significantly associated with gallstones were age (OR; 95% CI = 1.050; 1.042–1.058), family history of gallstones (OR; 95% CI = 1.683; 1.360–2.083), total bilirubin (OR; 95% CI = 1.823; 1.464–2.269), AST (OR; 95% CI = 0.988; 0.977–0.998), ALT (OR; 95% CI = 1.011; 1.005–1.0117), and HDL-C (OR; 95% CI = 0.985; 0.979–0.991). Additional file 1: Table S2 shows the risk of gallstones among individuals with the GC and CC genotypes compared to the GG genotype.

**Table 2:** Association between the ABCG8 rs11887534 variant and gallstones in the study participants

Variables	OR	95% CI		*P*-value
ABCG8 rs11887534 genotype				
GG (reference)	–	–	–	–
GC + CC	1.698	1.240	2.325	0.0010
Sex				
Women (reference)	–	–	–	–
Men	0.763	0.638	0.913	0.0032
Age	1.050	1.042	1.058	< .0001
Regular exercise				
No (reference)	–	–	–	–
Yes	0.885	0.769	1.020	0.0914
Cigarette smoking				
No (reference)	–	–	–	–
Yes	1.162	0.951	1.420	0.1430
Alcohol drinking				
No (reference)	–	–	–	–
Yes	0.997	0.772	1.289	0.9839
BMI				
Normal (reference)	–	–	–	–
Underweight	0.846	0.506	1.414	0.5238
Overweight	1.158	0.987	1.359	0.0721
Obesity	1.105	0.913	1.336	0.3052
Family history of gallstones				
No (reference)	–	–	–	–
Yes	1.683	1.360	2.083	< .0001
Diabetes				
No (reference)	–	–	–	–
Yes	1.128	0.927	1.373	0.2302
Total bilirubin	1.823	1.464	2.269	< .0001
AST	0.988	0.977	0.998	0.0214
ALT	1.011	1.005	1.017	0.0004
HDL-C (mg/dL)	0.985	0.979	0.991	< .0001
LDL-C (mg/dL)	0.998	0.996	1.000	0.1112
TG (mg/dL)	0.999	0.998	1.000	0.0593
Dietary fat score	1.000	0.992	1.007	0.8992

*OR* odds ratio, *CI* confidence interval, *ABCG8* ATP-binding cassette subfamily G Member 8, *BMI* body mass index, *AST* aspartate aminotransferase, *ALT* alanine transaminase, *HDL-C* high-density lipoprotein cholesterol, *LDL-C* low-density lipoprotein cholesterol, *TG* triglyceride

Table 3 shows the risk of gallstones according to the rs11887534 genotypes (GG and GC + CC) and sex. Compared to the reference group (men with the rs11887534 GG genotype), men with the GC + CC genotype had an elevated risk of gallstones but this observation did not achieve statistical significance (OR = 1.598, 95% CI = 0.941–2.714). However, women carrying both the rs11887534 GG and GC + CC genotypes had a significantly higher risk of gallstones. Of note, the risk was higher among those with the GC + CC genotype. The OR; 95% CI was 1.304; 1.087–1.565 for GG and 2.291; 1.514–3.467 for GC + CC. Age, family history, total bilirubin, AST, ALT, and HDL-C retained the significant relationship observed in the major effect model. The ORs; 95% CIs were 1.050; 1.042–1.058 for age, 1.683; 1.360–2.083 for family history of gallstones, 1.823; 1.464–2.269 for total bilirubin, 0.988; 0.977–0.998 for AST, 1.011; 1.005–1.0117 for ALT, and 0.985; 0.979–0.991 for HDL-C (Table 3). Additional file 1: Table S3 shows the risk of gallstones according to the rs11887534 genotypes (GG, GC, and CC) and sex.

**Table 3:** Association of the ABCG8 rs11887534 variant and sex with gallstones in the study participants

Variables	OR	95% CI		*P*-value
ABCG8 rs11887534 genotype, sex				
ABCG8 rs11887534 GG, men (reference)	–	–	–	–
ABCG8 rs11887534 GC + CC, men	1.598	0.941	2.714	0.0827
ABCG8 rs11887534 GG, women	1.304	1.087	1.565	0.0043
ABCG8 rs11887534 GC + CC, women	2.291	1.514	3.467	< .0001
Age	1.050	1.042	1.058	< .0001
Regular exercise				
No (reference)	–	–	–	–
Yes	0.885	0.769	1.020	0.0918
Cigarette smoking				
No (reference)	–	–	–	–
Yes	1.161	0.950	1.419	0.1448
Alcohol drinking				
No (reference)	–	–	–	–
Yes	0.997	0.771	1.288	0.9798
BMI				
Normal (reference)	–	–	–	–
Underweight	0.846	0.506	1.414	0.5239
Overweight	1.159	0.987	1.360	0.0712
Obesity	1.104	0.913	1.335	0.3057
Family history of gallstones				
No (reference)	–	–	–	–
Yes	1.683	1.360	2.083	< .0001
Diabetes				
No (reference)	–	–	–	–
Yes	1.128	0.927	1.373	0.2302
Total bilirubin	1.823	1.465	2.269	< .0001
AST	0.988	0.977	0.998	0.0213
ALT	1.011	1.005	1.017	0.0004
HDL-C (mg/dL)	0.985	0.979	0.991	< .0001
LDL-C (mg/dL)	0.998	0.996	1.000	0.1123
TG (mg/dL)	0.999	0.998	1.000	0.0592
Dietary fat score	1.000	0.992	1.007	0.8959

*OR* odds ratio, *CI* confidence interval, *ABCG8* ATP-binding cassette subfamily G Member 8, *BMI* body mass index, *AST* aspartate aminotransferase, *ALT* alanine transaminase, *HDL-C* high-density lipoprotein cholesterol, *LDL-C* low-density lipoprotein cholesterol, *TG* triglyceride

Further analysis including only women while considering hormonal and menopausal status retained the positive association of the GC + CC genotype with gallstones: OR; 95% CI = 1.789 = 1.208–2.648 (Table 4). Hormone use was associated with a higher risk of gallstones (OR; 95%CI = 1.359; 1.107–1.668). In this model, the previously observed relationship of gallstone with age, family history, total bilirubin, ALT, and HDL-C was retained. Exercise and overweight were respectively associated with a lower and higher risk of gallstones (OR; 95% CI = 0.816; 0.682–0.976 for exercise and 1.307; 1.069–1.598 for overweight). Additional file 1: Table S4 shows the risk of gallstones among women with the GC and CC genotypes compared to the GG genotype.

**Table 4:** Association between the ABCG8 rs11887534 variant and gallstones in the female study participants

Variables	OR	95% CI		*P*-value
ABCG8 rs11887534 genotype				
GG (reference)	–	–	–	–
GC + CC	1.789	1.208	2.648	0.0037
Hormone use				
No (reference)	–	–	–	–
Yes	1.359	1.107	1.668	0.0033
Menopause				
No (reference)	–	–	–	–
Yes	1.250	0.934	1.672	0.1332
Age	1.042	1.027	1.058	< .0001
Regular exercise				
No (reference)	–	–	–	–
Yes	0.816	0.682	0.976	0.0260
Cigarette smoking				
No (reference)	–	–	–	–
Yes	1.243	0.827	1.869	0.2963
Alcohol drinking				
No (reference)	–	–	–	–
Yes	1.317	0.786	2.205	0.2955
BMI				
Normal (reference)	–	–	–	–
Underweight	0.900	0.518	1.565	0.7099
Overweight	1.307	1.069	1.598	0.0090
Obesity	1.189	0.931	1.520	0.1660
Family history of gallstones				
No (reference)	–	–	–	–
Yes	1.730	1.332	2.246	< .0001
Diabetes				
No (reference)	–	–	–	–
Yes	1.054	0.805	1.380	0.6998
Total bilirubin	1.875	1.348	2.606	0.0002
AST	0.986	0.972	1.001	0.0709
ALT	1.011	1.002	1.021	0.0215
HDL-C (mg/dL)	0.988	0.981	0.996	0.0020
LDL-C (mg/dL)	0.997	0.994	0.999	0.0125
TG (mg/dL)	1.000	0.999	1.001	0.7819
Dietary fat score	1.005	0.995	1.014	0.3421

*OR* odds ratio, *CI* confidence interval, *ABCG8* ATP-binding cassette subfamily G Member 8, *BMI* body mass index, *AST* aspartate aminotransferase, *ALT* alanine transaminase, *HDL-C* high-density lipoprotein cholesterol, *LDL-C* low-density lipoprotein cholesterol, *TG* triglyceride

Table 5 shows the risk of gallstones based on the combination of rs11887534 genotypes (GG and GC + CC) and hormone use. Compared to the reference group (rs11887534 GG and no hormone use), the risk of gout was significantly higher in all groups. The OR; 95% CI was 1.333; 1.082–1.643 among hormone users with the GG genotype and 1.644; 1.061–2.548 among non-hormone users with the GC + CC genotype. Remarkably, the highest odds ratio was observed in hormone users with the rs11887534 GC + CC genotype (OR; 95% CI = 3.596; 1.495 to 8.650). Age, exercise, overweight, family history, total bilirubin, ALT, and HDL remained significantly associated with gallstones. Additional file 1: Table S5 shows the risk of gallstones based on the combination of rs11887534 genotypes (GG, GC, and CC) and hormone use.

**Table 5:** Association of the ABCG8 rs11887534 variant and hormone use with gallstones in the female study participants

Variables	OR	95% CI		*P*-value
ABCG8 rs11887534 genotype, hormone use				
ABCG8 rs11887534 GG, no hormone use (reference)	–	–	–	–
ABCG8 rs11887534 GG, hormone use	1.333	1.082	1.643	0.0069
ABCG8 rs11887534 GC + CC, no hormone use	1.644	1.061	2.548	0.0261
ABCG8 rs11887534 GC + CC, hormone use	3.596	1.495	8.650	0.0043
Menopause				
No (reference)	–	–	–	–
Yes	1.251	0.935	1.674	0.1312
Age	1.042	1.027	1.058	< .0001
Regular exercise				
No (reference)	–	–	–	–
Yes	0.816	0.682	0.975	0.0256
Cigarette smoking				
No (reference)	–	–	–	–
Yes	1.237	0.823	1.861	0.3068
Alcohol drinking				
No (reference)	–	–	–	–
Yes	1.322	0.790	2.214	0.2885
BMI				
Normal (reference)	–	–	–	–
Underweight	0.900	0.518	1.564	0.7084
Overweight	1.312	1.073	1.605	0.0081
Obesity	1.189	0.930	1.519	0.1667
Family history of gallstones				
No (reference)	–	–	–	–
Yes	1.733	1.334	2.251	< .0001
Diabetes				
No (reference)	–	–	–	–
Yes	1.052	0.804	1.378	0.7115
Total bilirubin	1.884	1.355	2.619	0.0002
AST	0.986	0.972	1.001	0.0710
ALT	1.011	1.002	1.021	0.0214
HDL-C (mg/dL)	0.988	0.981	0.996	0.0019
LDL-C (mg/dL)	0.997	0.994	0.999	0.0120
TG (mg/dL)	1.000	0.999	1.001	0.7689
Dietary fat score	1.005	0.995	1.014	0.3356

*OR* odds ratio, *CI* confidence interval, *ABCG8* ATP-binding cassette subfamily G Member 8, *BMI* body mass index, *AST* aspartate aminotransferase, *ALT* alanine transaminase, *HDL-C* high-density lipoprotein cholesterol, *LDL-C* low-density lipoprotein cholesterol, *TG* triglyceride

## Discussion

An in-depth understanding of population-specific risk factors, particularly genetic determinants of diseases, is important for the development of personalized preventive treatment options. In the present study, we assessed the association between gallstones and rs11887534 single nucleotide polymorphism, taking sex and hormone use into account. The GC + CC genotype of the variant was significantly associated with a higher risk of gallstones, especially among women. Because sex appeared to play a role in the association between the genetic variant and gallstones, we restricted the analysis to female participants and considered menopausal status and hormone use. Hormone use was significantly associated with a higher risk of gallstones. Further analysis taking into consideration hormone use and genotype showed that female hormone users with the GC + CC genotype had the highest risk of gallstones. In general, age, family history, total bilirubin, ALT, and HDL each had a significant association with gallstones irrespective of sex and hormone use habit.

The ABCG8 gene is highly expressed in gallbladder epithelial cells [36]. Genetic variation in this gene is associated with cardiovascular disease, gallstones, and cancer of the biliary tract [17, 19, 20, 28, 37–39]. The role of rs11887534, the most common genetic determinant for gallstone predisposition is well-evidenced from GWAS [22, 23] and several replication studies [7, 22, 26–30]. Replication studies involving Germans, Chileans, Chinese, Danish, Indians, African and Hispanic Americans all showed significant associations between rs11887534 and gallstones [22, 31, 40]. However, in a study involving Mexican Americans and non-Hispanic blacks and whites, significant associations were observed only among non-Hispanic whites. The non-significant results among Mexican Americans and non-Hispanic blacks were probably due to smaller sample sizes; 1705 Mexican Americans (227 cases and 1478 controls) and 1719 non-Hispanic blacks (179 cases and 1540) compared to 2408 Hispanic whites comprising 446 cases and 1962 controls [10].

Although several studies have been conducted on gallstones in Taiwan, only a few of them considered the role of the ABCG8 variant. In one study, Taiwanese with the GC genotype had higher levels of cholesterol and LDL [41]. In another study, the genotype was associated with a higher risk of gallstones [7]. The current study with a relatively large sample size further confirms the role of ABCG8 in gallstone predisposition among Taiwanese.

In mice and humans, this polymorphism promoted gallstone formation by increasing the expression of ABCG8, consequently enhancing biliary cholesterol production and buildup while decreasing dietary cholesterol absorption [40, 42, 43]. The decrease in dietary cholesterol absorption could be compensated for by high hepatic cholesterol synthesis leading to cholesterol-supersaturated bile.

Gallstone is predominant in women than men [8, 9, 12, 27, 44–46]. Exogenous female sex hormones are among the key players in the etiology of gallstones in women [11, 12]. One pathological mechanism is that female sex hormones alter cholesterol metabolism and gallbladder/intestinal motility. That is, they increase biliary cholesterol and alter bile composition, thereby impairing gallbladder motility [47]. In the liver, hormone replacement therapy, as well as the 17β-estradiol binding to estrogen receptors, promotes the release and saturation of cholesterol in bile [48, 49].

In a study among Chinese, the rs11887534 GC variant was associated with a higher probability of bladder cancer in both men and women, but significant results were prominent only among women [28]. Similarly, in a study among Indians, the association of gallstone with the ABCG8 rs11887534 polymorphism was more prominent in women [38]. The observation was attributed to hormone use [38]. In the current study, hormone use was associated with a higher risk of gallstones. Consistently, several studies reported a higher risk of gallbladder diseases with higher contraceptive use and hormone use [12–16, 46, 50–53]. Considering both sex and genotype in the current study, we found no significant risk of gallstone in men irrespective of the genotype. Interestingly, the highest OR for gallstone was reported among female hormone users carrying the GC + CC genotype. These results imply that the role of rs11887534 in gallstones might be more prominent in women with the strongest effect in hormone users, further confirming the role of hormone use in gallstone etiology.

Age and BMI are also independent determinants of gallstones [6, 8, 9, 27, 45, 46, 52–58]. Other reported risk factors for gallstones are family history [6], abnormal liver function tests including high bilirubin, AST, and ALT [54–56, 59], and abnormal lipoprotein levels including low HDL-C, high LDL-C, and triglycerides [46, 58, 60]. In the current study, age, family history, total bilirubin, AST, ALT, and HDL-C HDL were consistently associated with the risk of gallstone. Even though BMI was a risk factor, its association with gallstone was prominent only when we restricted the analysis to only female participants. Regular exercise was not significantly associated with the risk of gallstones in the general participants, but a sub-analysis involving only women showed a significant inverse association. This observation has been previously reported in both men and women [53, 61, 62] and women [57, 63].

As a strength, this nationwide population study among Taiwanese adults included 21,268 individuals (938 cases and 20,330 controls). This is a very large sample size compared to several studies. For instance, the only study in Taiwan that assessed the association between gallstones and rs11887534 polymorphism had only 979 participants [7]. Moreover, a study replicating the association of gallstones with 11,887,534 included only 334 Chileans (167 cases and 167 controls) and 1470 Germans comprising 728 cases and 732 controls [22]. Another replication study included only 2408 non-Hispanic whites, 1705 Mexican Americans, and 1719 non-Hispanic blacks [10]. In another study, there were 1122 Chileans (680 cases and 442 controls), 1132 Danish (366 cases and 766 controls), 471 Indians (247 cases and 224 controls), and 524 Chinese (280 cases and 244 controls [40].

As a limitation, gallstone information in this study was obtained through questionnaires which could have increased the probability of information bias. Moreover, we did not stratify the participants based on the specific purpose for which they used hormones. Hence, the use of oral contraceptives and hormone replacement therapy were all considered as hormone use.

## Conclusions

The GC + CC genotype was independently associated with a higher risk of gallstones. Having the genotype and being a female participant was associated with a much-elevated risk. Hormone use among women with the GC + CC genotype was also associated with a much higher risk. This suggests that genetic factors and female hormones are very substantial in the etiology of gallstones and a combination of both factors could exacerbate the disease risk.

## Supplementary Information

**Additional file 1**. **Table S1.** Baseline profiles of participants stratified by sex showing the frequency of the GG, GC, and CC genotypes. **Table S2.** Risk of gallstones among individuals with the GC and CC genotypes compared to the GG genotype. **Table S3.** Risk of gallstones according to the rs11887534 genotypes (GG, GC, and CC) and sex. **Table S4.** Risk of gallstones among women with the GC and CC genotypes compared to the GG genotype. **Table S5.** Risk of gallstones based on the combination of rs11887534 genotypes (GG, GC, and CC) and hormone use.

## Data Availability

The data that support the findings of this study are available from Taiwan Biobank but restrictions apply to the availability of these data, which were used under license for the current study, and so are not publicly available. Data are however available from the authors upon reasonable request and with permission of Taiwan Biobank.
