# Supplementary material for: Risk of gallstones based on ABCG8 rs11887534 single nucleotide polymorphism among Taiwanese men and women
Source: BMC Gastroenterol. 2021 Dec 14;21:468. doi: 10.1186/s12876-021-02060-5 (PMC8672562; doi:10.1186/s12876-021-02060-5)
Supplement: Supplementary file 1 — Additional file 1. Table S1. Baseline profiles of participants stratified by sex showing the frequency of the GG, GC, and CC genotypes. Table S2. Risk of gallstones among individuals with the GC and CC genotypes compared to the GG genotype. Table S3. Risk of gallstones according to the rs11887534 genotypes (GG, GC, and CC) and sex. Table S4. Risk of gallstones among women with the GC and CC genotypes compared to the GG genotype. Table S5. Risk of gallstones based on the combination of rs11887534 genotypes (GG, GC, and CC) and hormone use. [file 12876_2021_2060_MOESM1_ESM.docx]

Supplementary Table 1. Baseline profiles of participants stratified by sex showing the frequency of the GG, GC, and CC genotypes.

| Variables | Women (n = 13880) | Men (n = 7388) | P-value |
| --- | --- | --- | --- |
| Gallstone |  |  | 0.1043 |
| No | 13291 (95.76) | 7039 (95.28) |  |
| Yes | 589 (4.24) | 349 (4.72) |  |
| ABCG8 rs11887534 genotype |  |  | 0.7495 |
| GG | 13462 (96.99) | 7178 (97.16) |  |
| GC | 415 (2.99) | 208 (2.82) |  |
| CC | 3 (0.02) | 2 (0.03) |  |
| Age (years) | 49.7555±0.088 | 49.8679±0.128 | 0.4702 |
| Regular exercise |  |  | 0.0023 |
| No | 8032 (57.87) | 4115 (55.70) |  |
| Yes | 5848 (42.13) | 3273 (44.30) |  |
| Cigarette smoking |  |  | <.0001 |
| No | 13272 (95.62) | 4149 (56.16) |  |
| Yes | 608 (4.38) | 3239 (43.84) |  |
| Alcohol drinking |  |  | <.0001 |
| No | 13571 (97.77) | 5992 (81.10) |  |
| Yes | 309 (2.23) | 1396 (18.90) |  |
| BMI (kg/m^2^) |  |  | <.0001 |
| 18.5≤BMI<24 (Normal BMI) | 7944 (57.23) | 2666 (36.09) |  |
| BMI<18.5 (Underweight) | 531 (3.83) | 83 (1.12) |  |
| 24≤BMI<27 (Overweight) | 3280 (23.63) | 2698 (36.52) |  |
| BMI≥27 (Obesity) | 2125 (15.31) | 1941 (26.27) |  |
| Family history of gallstones |  |  | 0.0153 |
| No | 12812 (92.31) | 6887 (93.22) |  |
| Yes | 1068 (7.69) | 501 (6.78) |  |
| Diabetes |  |  | <.0001 |
| No | 12759 (91.92) | 6390 (86.49) |  |
| Yes | 1121 (8.08) | 998 (13.51) |  |
| Total bilirubin (mg/dL) | 0.6138±0.002 | 0.7516±0.004 | <.0001 |
| AST (U/L) | 23.1276±0.100 | 26.2884±0.173 | <.0001 |
| ALT (U/L) | 20.8680±0.147 | 29.9571±0.274 | <.0001 |
| HDL-C (mg/dL) | 58.3463±0.113 | 48.1481±0.131 | <.0001 |
| LDL-C (mg/dL) | 120.7000±0.271 | 122.3000±0.368 | 0.0003 |
| TG (mg/dL) | 102.3000±0.608 | 137.000±1.288 | <.0001 |
| Dietary fat score | 41.7411±0.078 | 45.9518±0.111 | <.0001 |
| Menopause |  |  |  |
| No | 6937 (49.98) | - | - |
| Yes | 6943 (50.02) | - | - |
| Hormone use |  |  |  |
| No | 11721 (84.45) | - | - |
| Yes | 2159 (15.55) | - | - |

n= sample size, ABCG8 = ATP-binding cassette subfamily G Member 8, BMI = body mass index, AST = aspartate aminotransferase, ALT = alanine transaminase, HDL-C = high-density lipoprotein cholesterol, LDL-C = low-density lipoprotein cholesterol, TG = triglyceride.

Continuous and categorical data are presented as mean±standard error (SE) and n (%), respectively.

Supplementary Table 2. Risk of gallstones among individuals with the GC and CC genotypes compared to the GG genotype.

| Variables | OR |  | 95 % CI | | P-value |
| --- | --- | --- | --- | --- | --- |
| ABCG8 rs11887534 genotype |  |  |  |  |  |
| GG (reference) | - |  | - | - | - |
| GC | 1.722 |  | 1.257 | 2.359 | 0.0007 |
| CC | <0.001 |  | <0.001 | >999.999 | 0.9597 |
| Sex |  |  |  |  |  |
| Women (reference) | - |  | - | - | - |
| Men | 0.763 |  | 0.638 | 0.913 | 0.0032 |
| Age | 1.050 |  | 1.042 | 1.058 | <.0001 |
| Regular exercise |  |  |  |  |  |
| No (reference) | - |  | - | - | - |
| Yes | 0.886 |  | 0.769 | 1.021 | 0.0935 |
| Cigarette smoking |  |  |  |  |  |
| No (reference) | - |  | - | - | - |
| Yes | 1.161 |  | 0.950 | 1.419 | 0.1451 |
| Alcohol drinking |  |  |  |  |  |
| No (reference) | - |  | - | - | - |
| Yes | 0.997 |  | 0.771 | 1.288 | 0.9807 |
| BMI |  |  |  |  |  |
| Normal (reference) | - |  | - | - | - |
| Underweight | 0.847 |  | 0.507 | 1.414 | 0.5247 |
| Overweight | 1.159 |  | 0.988 | 1.360 | 0.0700 |
| Obesity | 1.104 |  | 0.913 | 1.335 | 0.3076 |
| Family history of gallstones |  |  |  |  |  |
| No (reference) | - |  | - | - | - |
| Yes | 1.682 |  | 1.359 | 2.081 | <.0001 |
| Diabetes |  |  |  |  |  |
| No (reference) | - |  | - | - | - |
| Yes | 1.129 |  | 0.927 | 1.374 | 0.2276 |
| Total bilirubin | 1.822 |  | 1.464 | 2.268 | <.0001 |
| AST | 0.988 |  | 0.977 | 0.998 | 0.0212 |
| ALT | 1.011 |  | 1.005 | 1.017 | 0.0005 |
| HDL-C (mg/dL) | 0.985 |  | 0.978 | 0.991 | <.0001 |
| LDL-C (mg/dL) | 0.998 |  | 0.996 | 1.000 | 0.1110 |
| TG (mg/dL) | 0.999 |  | 0.998 | 1.000 | 0.0599 |
| Dietary fat score | 1.000 |  | 0.992 | 1.007 | 0.9075 |

OR = odds ratio, CI = confidence interval, ABCG8 = ATP-binding cassette subfamily G Member 8, BMI = body mass index, AST = aspartate aminotransferase, ALT = alanine transaminase, HDL-C = high-density lipoprotein cholesterol, LDL-C = low-density lipoprotein cholesterol, TG = triglyceride.

Supplementary Table 3. Risk of gallstones according to the rs11887534 genotypes (GG, GC, and CC) and sex.

| Variables | OR |  | 95 % CI | | P-value |
| --- | --- | --- | --- | --- | --- |
| ABCG8 rs11887534 genotype, sex |  |  |  |  |  |
| ABCG8 rs11887534 GG, men (reference) | - |  | - | - | - |
| ABCG8 rs11887534 GC, men | 1.628 |  | 0.959 | 2.766 | 0.0713 |
| ABCG8 rs11887534 CC, men | <0.001 |  | <0.001 | >999.999 | 0.9743 |
| ABCG8 rs11887534 GG, women | 1.305 |  | 1.087 | 1.566 | 0.0043 |
| ABCG8 rs11887534 GC, women | 2.318 |  | 1.532 | 3.509 | <.0001 |
| ABCG8 rs11887534 CC, women | <0.001 |  | <0.001 | >999.999 | 0.9705 |
| Age | 1.050 |  | 1.042 | 1.058 | <.0001 |
| Regular exercise |  |  |  |  |  |
| No (reference) | - |  | - | - | - |
| Yes | 0.886 |  | 0.769 | 1.021 | 0.0938 |
| Cigarette smoking |  |  |  |  |  |
| No (reference) | - |  | - | - | - |
| Yes | 1.160 |  | 0.949 | 1.419 | 0.1466 |
| Alcohol drinking |  |  |  |  |  |
| No (reference) | - |  | - | - | - |
| Yes | 0.996 |  | 0.771 | 1.288 | 0.9769 |
| BMI |  |  |  |  |  |
| Normal (reference) | - |  | - | - | - |
| Underweight | 0.847 |  | 0.507 | 1.414 | 0.5248 |
| Overweight | 1.160 |  | 0.988 | 1.361 | 0.0691 |
| Obesity | 1.104 |  | 0.913 | 1.335 | 0.3081 |
| Family history of gallstones |  |  |  |  |  |
| No (reference) | - |  | - | - | - |
| Yes | 1.682 |  | 1.359 | 2.082 | <.0001 |
| Diabetes |  |  |  |  |  |
| No (reference) | - |  | - | - | - |
| Yes | 1.129 |  | 0.927 | 1.374 | 0.2276 |
| Total bilirubin | 1.823 |  | 1.464 | 2.269 | <.0001 |
| AST | 0.988 |  | 0.977 | 0.998 | 0.0211 |
| ALT | 1.011 |  | 1.005 | 1.017 | 0.0004 |
| HDL-C (mg/dL) | 0.985 |  | 0.978 | 0.991 | <.0001 |
| LDL-C (mg/dL) | 0.998 |  | 0.996 | 1.000 | 0.1120 |
| TG (mg/dL) | 0.999 |  | 0.998 | 1.000 | 0.0599 |
| Dietary fat score | 1.000 |  | 0.992 | 1.007 | 0.9044 |

OR = odds ratio, CI = confidence interval, ABCG8 = ATP-binding cassette subfamily G Member 8, BMI = body mass index, AST = aspartate aminotransferase, ALT = alanine transaminase, HDL-C = high-density lipoprotein cholesterol, LDL-C = low-density lipoprotein cholesterol, TG = triglyceride.

Supplementary Table 4. Risk of gallstones among women with the GC and CC genotypes compared to the GG genotype

| Variables | OR |  | 95 % CI | | P-value |
| --- | --- | --- | --- | --- | --- |
| ABCG8 rs11887534 genotype |  |  |  |  |  |
| GG (reference) | - |  | - | - | - |
| GC | 1.809 |  | 1.222 | 2.679 | 0.0031 |
| CC | <0.001 |  | <0.001 | >999.999 | 0.9700 |
| Hormone use |  |  |  |  |  |
| No (reference) | - |  | - | - | - |
| Yes | 1.358 |  | 1.106 | 1.666 | 0.0034 |
| Menopause |  |  |  |  |  |
| No (reference) | - |  | - | - | - |
| Yes | 1.250 |  | 0.935 | 1.672 | 0.1326 |
| Age | 1.042 |  | 1.027 | 1.058 | <.0001 |
| Regular exercise |  |  |  |  |  |
| No (reference) | - |  | - | - | - |
| Yes | 0.817 |  | 0.683 | 0.977 | 0.0264 |
| Cigarette smoking |  |  |  |  |  |
| No (reference) | - |  | - | - | - |
| Yes | 1.246 |  | 0.828 | 1.873 | 0.2912 |
| Alcohol drinking |  |  |  |  |  |
| No (reference) | - |  | - | - | - |
| Yes | 1.316 |  | 0.786 | 2.204 | 0.2968 |
| BMI |  |  |  |  |  |
| Normal (reference) | - |  | - | - | - |
| Underweight | 0.901 |  | 0.518 | 1.565 | 0.7103 |
| Overweight | 1.308 |  | 1.070 | 1.599 | 0.0088 |
| Obesity | 1.188 |  | 0.930 | 1.519 | 0.1678 |
| Family history of gallstones |  |  |  |  |  |
| No (reference) | - |  | - | - | - |
| Yes | 1.729 |  | 1.331 | 2.245 | <.0001 |
| Diabetes |  |  |  |  |  |
| No (reference) | - |  | - | - | - |
| Yes | 1.055 |  | 0.806 | 1.381 | 0.6968 |
| Total bilirubin | 1.872 |  | 1.347 | 2.603 | 0.0002 |
| AST | 0.986 |  | 0.972 | 1.001 | 0.0708 |
| ALT | 1.011 |  | 1.002 | 1.021 | 0.0217 |
| HDL-C (mg/dL) | 0.988 |  | 0.981 | 0.996 | 0.0019 |
| LDL-C (mg/dL) | 0.997 |  | 0.994 | 0.999 | 0.0124 |
| TG (mg/dL) | 1.000 |  | 0.999 | 1.001 | 0.7847 |
| Dietary fat score | 1.005 |  | 0.995 | 1.014 | 0.3384 |

OR = odds ratio, CI = confidence interval, ABCG8 = ATP-binding cassette subfamily G Member 8, BMI = body mass index, AST = aspartate aminotransferase, ALT = alanine transaminase, HDL-C = high-density lipoprotein cholesterol, LDL-C = low-density lipoprotein cholesterol, TG = triglyceride.

Supplementary Table 5. Risk of gallstones based on the combination of rs11887534 genotypes (GG, GC, and CC) and hormone use.

| Variables | OR | 95 % CI | | P-value |
| --- | --- | --- | --- | --- |
| ABCG8 rs11887534 genotype, hormone use |  |  |  |  |
| ABCG8 rs11887534 GG, no hormone use (reference) | - | - | - | - |
| ABCG8 rs11887534 GG, hormone use | 1.333 | 1.082 | 1.643 | 0.0070 |
| ABCG8 rs11887534 GC, no hormone use | 1.667 | 1.075 | 2.583 | 0.0224 |
| ABCG8 rs11887534 GC, hormone use | 3.594 | 1.494 | 8.646 | 0.0043 |
| ABCG8 rs11887534 CC, no hormone use | <0.001 | <0.001 | >999.999 | 0.9700 |
| ABCG8 rs11887534 CC, hormone use | - | - | - | - |
| Menopause |  |  |  |  |
| No (reference) | - | - | - | - |
| Yes | 1.252 | 0.936 | 1.674 | 0.1308 |
| Age | 1.043 | 1.027 | 1.058 | <.0001 |
| Regular exercise |  |  |  |  |
| No (reference) | - | - | - | - |
| Yes | 0.816 | 0.682 | 0.976 | 0.0260 |
| Cigarette smoking |  |  |  |  |
| No (reference) | - | - | - | - |
| Yes | 1.240 | 0.824 | 1.865 | 0.3017 |
| Alcohol drinking |  |  |  |  |
| No (reference) | - | - | - | - |
| Yes | 1.321 | 0.789 | 2.212 | 0.2898 |
| BMI |  |  |  |  |
| Normal (reference) | - | - | - | - |
| Underweight | 0.900 | 0.518 | 1.564 | 0.7088 |
| Overweight | 1.313 | 1.074 | 1.606 | 00080 |
| Obesity | 1.188 | 0.930 | 1.518 | 0.1684 |
| Family history of gallstones |  |  |  |  |
| No (reference) | - | - | - | - |
| Yes | 1.732 | 1.334 | 2.249 | <.0001 |
| Diabetes |  |  |  |  |
| No (reference) | - | - | - | - |
| Yes | 1.053 | 0.804 | 1.378 | 0.7085 |
| Total bilirubin | 1.881 | 1.353 | 2.615 | 0.0002 |
| AST | 0.986 | 0.972 | 1.001 | 0.0709 |
| ALT | 1.011 | 1.002 | 1.021 | 0.0215 |
| HDL-C (mg/dL) | 0.988 | 0.981 | 0.996 | 0.0019 |
| LDL-C (mg/dL) | 0.997 | 0.994 | 0.999 | 0.0120 |
| TG (mg/dL) | 1.000 | 0.999 | 1.001 | 0.7718 |
| Dietary fat score | 1.005 | 0.995 | 1.014 | 0.3323 |

OR = odds ratio, CI = confidence interval, ABCG8 = ATP-binding cassette subfamily G Member 8, BMI = body mass index, AST = aspartate aminotransferase, ALT = alanine transaminase, HDL-C = high-density lipoprotein cholesterol, LDL-C = low-density lipoprotein cholesterol, TG = triglyceride.
